# TGF-β promotes pericyte-myofibroblast transition in subretinal fibrosis through the Smad2/3 and Akt/mTOR pathways

**DOI:** 10.1038/s12276-022-00778-0

**Published:** 2022-05-27

**Authors:** Zhenzhen Zhao, Yumeng Zhang, Chaoyang Zhang, Jingting Zhang, Xueting Luo, Qinghua Qiu, Dawei Luo, Jingfa Zhang

**Affiliations:** 1 Department of Ophthalmology, Shanghai General Hospital (Shanghai First People’s Hospital), Shanghai Jiao Tong University, School of Medicine, Shanghai, China; 2 National Clinical Research Center for Eye Diseases; Shanghai Key Laboratory of Ocular Fundus Diseases; Shanghai Engineering Center for Visual Science and Photomedicine; Shanghai Engineering Center for Precise Diagnosis and Treatment of Eye Diseases, Shanghai, China; 3 Department of Ophthalmology, Shigatse People’s Hospital, Xizang, China

**Keywords:** Growth factor signalling, Transforming growth factor beta

## Abstract

Subretinal fibrosis remains a major obstacle to the management of neovascular age-related macular degeneration. Choroidal pericytes were found to be a significant source of subretinal fibrosis, but the underlying mechanisms of pericyte-myofibroblast transition (PMT) remain largely unknown. The goal of this study was to explore the role and potential mechanisms by which PMT contributes to subretinal fibrosis. Choroidal neovascularization (CNV) was induced by laser photocoagulation in transgenic mice with the collagen1α1-green fluorescent protein (Col1α1-GFP) reporter, and recombinant adeno-associated virus 2 (rAAV2)-mediated TGF-β2 (rAAV2-TGF-β2) was administered intravitreally to further induce PMT. Primary mouse choroidal GFP-positive pericytes were treated with TGF-β2 in combination with siRNAs targeting Smad2/3, the Akt inhibitor MK2206 or the mTOR inhibitor rapamycin to examine cell proliferation, migration, and differentiation into myofibroblasts. The involvement of the Akt/mTOR pathway in PMT in subretinal fibrosis was further investigated in vivo. Intraocular TGF-β2 overexpression induced GFP-positive pericyte infiltration and PMT in subretinal fibrosis, which was mimicked in vitro. Knockdown of Smad2/3 or inhibition of Akt/mTOR decreased cell proliferation, PMT and migration in primary mouse pericytes. Combined inhibition of Smad2/3 and mTOR showed synergistic effects on attenuating α-smooth muscle actin (α-SMA) expression and cell proliferation. In mice with laser-induced CNV, the administration of the Akt/mTOR inhibitors suppressed pericyte proliferation and alleviated the severity of subretinal fibrosis. Our results showed that PMT plays a pivotal role in subretinal fibrosis, which was induced by TGF-β2 through the Smad2/3 and Akt/mTOR pathways. Thus, inhibiting PMT may be a novel strategy for the treatment of subretinal fibrosis.

## Eye disease: Revealing the mechanisms behind subretinal fibrosis

The identification of a new cell type that plays a crucial role in causing fibrosis under the retina could improve treatment of eye disease. Effective treatments exist for diseases that cause impairment and loss of vision in elderly people, but success can be limited by the development of subretinal fibrosis. Jingfa Zhang at Shanghai Jiao Tong University, China, and co-workers used mice with laser-induced retinal damage to explore how subretinal fibrosis may result from transition of pericytes, multi-functional cells in the capillaries, into myofibroblasts, cells associated with fibrosis. The overexpression of a growth factor called TGF-β2 induced pericytes to infiltrate the subretinal area and pericyte-myofibroblast transition via two signalling pathways. Inhibiting these pathways may help to treat subretinal fibrosis, and one option is the use of inhibitors of AKT/mTOR which may slow the ageing process.

## Introduction

Age-related macular degeneration (AMD) is a leading cause of visual impairment and severe vision loss that affects elderly individuals worldwide^1,2^. Its wet form, also known as macular neovascularization (MNV)^3^, accounts for approximately 10−15% of AMD cases, is mainly characterized by choroidal neovascularization (CNV) and progresses rapidly to cause severe vision loss and eventual subretinal fibrosis if left untreated^2^. Vascular endothelial growth factor (VEGF)-A plays distinct roles in pathological angiogenesis and inflammation in AMD, and anti-VEGF therapy has become the first-line treatment of MNV^4,5^. However, a substantial portion of patients still suffer from poor visual prognosis due to the development of subretinal fibrosis even with intensive anti-VEGF treatment^6^.

Subretinal fibrosis remains a major obstacle for MNV management, and there are currently no effective therapeutic interventions^7^. During MNV progression, it develops from neovascular membranes to a variably mixed fibrovascular structure, such as fibrosis containing infiltrating immune cells, myofibroblasts and excessive amounts of extracellular matrix (ECM) proteins, which eventually forms scars and is resistant to anti-VEGF treatment^7,8^. Myofibroblasts are fibroblast-like cells that express α-smooth muscle actin (α-SMA), deposit pathological ECM, and are the main factors in subretinal fibrosis^9^. It has been hypothesized that myofibroblasts in macular fibrosis originate from the differentiation/transition of multiple cell types, such as retinal pigment epithelial (RPE) cells, macrophages, and endothelial cells, although direct evidence in patients is still lacking^10–12^.

In our previous study, choroid-derived pericytes infiltrated the subretinal space, contributing to ECM deposition and fibrosis formation in a mouse model of laser-induced CNV^13^. In other organs, including the kidney, lung and spinal cord, pericytes have been shown to function as the primary fibrosis-forming cells and are the key contributors to fibrosis^14–16^. A recent study showed that inhibiting pericytes by blocking PDGFRβ signaling suppressed the formation of CNV and subretinal fibrosis in mice with laser-induced CNV^17^, indicating the involvement of pericytes in fibrosis formation. We therefore hypothesized that in the laser-induced CNV model, choroidal pericytes might differentiate into myofibroblast-like cells, which play a pivotal role in the formation and development of subretinal fibrosis. Transforming growth factor-beta (TGF-β) is extensively implicated in the pathogenesis of various types of fibrosis, including subretinal fibrosis, while TGF-β2 was shown to be more highly expressed than other TGF-β isoforms^7,12,18,19^. As a common mechanism of the mesenchymal transition during fibrosis, such as the transition of RPE cells into myofibroblasts^20^, TGF-β signaling pathways, which consist of TGF-β/Smad canonical signaling and non-Smad signaling pathways, promote fibrosis^21^. Although pericytes have been revealed to transdifferentiate into myofibroblasts in response to TGF-β2 stimulation in other organs, such as the lung^22^, the contribution of choroidal pericyte-myofibroblast transition (PMT) to subretinal fibrosis and the detailed mechanisms in response to TGF-β2 in a laser-induced CNV model remain unknown.

In this study, we performed both in vivo and in vitro experiments to investigate the effect of TGF-β2 on PMT and the possible mechanisms in the pathogenesis of subretinal fibrosis. We used transgenic mice in which GFP expression was driven by the collagen type 1 promoter (Col1α1-GFP mice) as a valuable tool to determine the molecular and cellular mechanisms of choroidal pericyte activation and subretinal fibrosis^13^. The results showed that TGF-β2 mediated choroidal pericyte proliferation and PMT via the Smad2/3 and Akt/mTOR signaling pathways, and blocking the Akt/mTOR pathway alleviated subretinal fibrosis in a laser-induced CNV model.

## Materials and methods

### Animals

C57BL/6 J mice or Col1α1-GFP transgenic mice aged 2–3 months were used in this study. Col1α1-GFP transgenic mice were generated as previously described^23^. Animal procedures conformed with the National Institutes of Health (USA) Guide for the Care and Use of Laboratory Animals (NIH Publication No. 8023, revised 1978) and were approved by the Shanghai Jiao Tong University Institutional Review Board.

### Laser-induced CNV mouse model

Laser-induced CNV was performed as described previously^13^. Briefly, mice (7–8 weeks old) were anesthetized with sodium pentobarbital, and the pupils were dilated with 1% tropicamide (Santen, Osaka, Japan). The four injury spots were induced by a 532-nm laser with a power of 120 mW for 0.1 seconds (Visulas 532 S; Carl Zeiss Meditec, Dublin, Ireland) in a manner fashion around the optic nerve using a slit-lamp system.

### Intravitreal injections

To examine the effect of TGF-β2 on choroidal pericytes in vivo, 2 weeks before laser injury, Col1α1-GFP transgenic mice received an intravitreal injection of rAAV2-TGF-β2 (1 µL, 1 × 10^8^ vg) or an equivalent volume of normal saline. The mice were sacrificed 3 weeks after laser-induced CNV. The sizes of fibrotic lesions were determined by measuring the areas of GFP-positive lesions and α-SMA-positive lesions on RPE-choroid complexes. Moreover, to exclude the effect of rAAV2 vector administration, C57BL/6 J mice were injected intravitreally with the same dose of the rAAV2 vector or 1 µL normal saline 2 weeks before laser injury and sacrificed 3 weeks after laser induction. The sizes of fibrotic lesions were determined by measuring the areas of PDGFRβ-positive and α-SMA-positive lesions on RPE-choroid complexes. For inhibitor administration, C57BL/6 J mice were injected intravitreally with Akt/mTOR inhibitors immediately after laser injury and 1 week later for early event analysis or at 2 weeks after injury to examine late events. The mice were divided into the following groups: (1) the MK2206 (10 mM × 2 μL/eye, Selleck Chemicals, Houston, TX, USA) group; (2) the rapamycin (500 μM × 2 μL/eye, Selleck Chemicals) group; (3) the dimethyl sulfoxide (DMSO) control (2 μL/eye) group; and (4) the normal untreated control group. The mice were sacrificed 3 weeks after laser injury, and subretinal fibrosis was analyzed.

### Isolation, identification and culture of primary choroidal GFP-positive pericytes

RPE/choroid tissue was incubated with collagenase A (6.25 mg/mL, Roche, Basel, Switzerland), dispase II (6.25 mg/mL, Roche), and DNase (62.5 µg/mL, Roche) at 37 °C for 15 min as previously described^24^ and then incubated in 0.25% trypsin-EDTA (Thermo Fisher Scientific, Waltham, MA, USA) at 37 °C for 5 min to dissociate single cells. The cell suspension was seeded in a 24-well plate containing MEM α modification (Thermo Fisher Scientific), 1% GlutaMAX (Thermo Fisher Scientific), 20% FBS (Sigma–Aldrich, St. Louis, MO, USA) and 1% penicillin/streptomycin (Thermo Fisher Scientific). The cells were identified with appropriate markers, such as PDGFRβ (+) and CD31 (−), to confirm the isolation of primary pericytes (Fig. 2a, Supplementary Fig. 2). The cells were passaged and cultured for 3 to 8 passages and used for further experiments.

### CCK8 assay

Cell proliferation was assessed with Cell Counting Kit‐8 (CCK8 assay; Sigma–Aldrich) according to the manufacturer’s instructions. Cells were seeded in 96-well plates at a density of 1,000 cells/well, and CCK8 solution (10 μL) was added to each well and incubated for 2 h. The cell proliferation curves were plotted after the absorbance was measured at 450 nm.

### Cell counting

Cell proliferation was measured by counting the number of cells. Cells were seeded at a density of 5 × 10^4^ cells/well in six-well plates and grown in complete medium for 12 h. Then, the cells were starved in 1% FBS low serum medium overnight and were pretreated before being stimulated with TGF-β2 (10 ng/mL, R&D Systems, MN, USA) for 1 day. The experiment was repeated three times.

### EdU assay

To further evaluate cell proliferation, an EdU assay was conducted using the BeyoClick™ EdU Cell Proliferation Kit with Alexa Fluor 594 (Beyotime, Shanghai, China), and Hoechst 33342 was used for nuclear staining according to the manufacturer’s instructions. Briefly, cells were incubated with EdU staining buffer for 8 h and fixed with 4% polyformaldehyde, and the nuclei were stained with Hoechst. The stained cells were photographed under a microscope. Cells that stained both red and blue were considered EdU-positive cells. Random fields were selected and imaged. Cells were counted using ImageJ software. The ratio of EdU-positive cells to total cells was calculated as the cell proliferation rate. The experiment was repeated three times.

### Transwell migration assay

Transwell migration assays were conducted after the cells were harvested with trypsin and resuspended (1 × 10^5^ cells/mL) in 1% FBS low serum medium. Cells were pretreated and subsequently stimulated with TGF-β2 (10 ng/mL) for 24 h. Complete medium was added to the bottom wells of the chambers. After being incubated at 37 °C with 5% CO_2_ for 24 h, cells that did not migrate were removed from the upper surface of the filters using cotton swabs, and the cells that had migrated to the opposite side of the transwell were fixed and stained with crystal violet solution. The number of cells was counted in three random fields under an inverted microscope, and the means were calculated. The experiments were repeated three times.

### Wound healing assay

A total of 2 × 10^5^ cells/well were plated and starved in 1% FBS low serum medium overnight. Then, scratches on the cell monolayers were made with a sterilized 1000-μL pipette tip. Images were recorded at the following time points: 0 h, 6 h, 12 h, 24 h, 36 h, 48 h, and 72 h. Wound recovery was analyzed using ImageJ software. The migration capacity was determined using the percentage of wound closure.

### RNA interference

Smad2 siRNA (si.Smad2) and Smad3 siRNAs(si.Smad3) were synthesized by Genomeditech Company (Shanghai, China). The sequences are shown in Supplementary Table 1. Smad2/3 siRNA (si.Smad2/3) was purchased from Santa Cruz Biotechnology (Dallas, TX, USA). The siRNAs were transfected with RFect Transfection Reagent (BAIDAI Biotech, Changzhou, China) according to the manufacturer’s instructions^25^. Briefly, to knockdown Smad2 or Smad3 individually or together, GFP-positive primary pericytes were treated with 30 nM siRNA and 0.5% RFect Transfection Reagent for 48 h. The transfection protocol used 30 nM si.Smad2/3 and 0.166% RFect Transfection Reagent in combination with different signaling pathways. After being transfected, the cells were cultured in 1% FBS media for 16 h and then split into different groups that were subjected to different treatments. The siRNA information is shown in Supplementary Table 1.

### Real-Time PCR

Total RNA was isolated according to the RNAsimple Total Kit protocol (Tiangen Biotech, Beijing, China) and quantified by a NanoDrop2000 spectrophotometer (Thermo Fisher Scientific). cDNA was synthesized according to the RT Master Mix protocol (Takara Bio Inc., Dalian, China). The primer information is shown in Supplementary Table 2.

### Western blotting

RPE-choroid complexes or cells were lysed in RIPA buffer (Beyotime) for protein extraction according to the manufacturer’s instructions. The protein concentration was measured with a Pierce bicinchoninic acid (BCA) assay (Thermo Fisher Scientific). Approximately 10 μg of total protein was added to SDS–PAGE gels and transferred onto a PVDF membrane (Merck Millipore, Billerica, MA, USA). The blots were blocked for 1 h in 5% nonfat milk in TBST (10 mM Tris, pH 8.0, 150 mM NaCl, 0.5% Tween 20) at room temperature and then incubated with primary antibodies overnight at 4 °C. The membranes were washed with TBST and incubated with secondary antibodies (1:5000, Proteintech, Wuhan, China) for 1 h. Then, the blots were exposed to a molecular imaging system (Amersham Imager 600, GE Healthcare, Buckinghamshire, UK). The antibody information is listed in Supplementary Table 3.

### Immunofluorescence analysis

Mice were killed and perfused intracardially with 4% paraformaldehyde in phosphate-buffered saline (PBS). The eyeballs were enucleated, retinal flatmounts were prepared, RPE-choroid complexes were flatmounted, and cryosections (10 μm) were prepared for immunofluorescence analysis. The samples were immunostained with antibodies against GFP, α-SMA, PDGFRβ, BEST1, CD31, and GS, respectively. The sizes of fibrotic lesions were determined using ImageJ, which measured the areas of GFP-positive lesions or PDGFRβ-positive and α-SMA-positive lesions on RPE-choroid complexes. Cells on cover slips were washed with 1× PBS and fixed in 4% paraformaldehyde in PBS for 20 min. After being fixed, the samples were blocked with 1× PBS containing 0.3% Triton X-100 and 5% goat serum albumin (Beyotime) for 1 h. Then, the cells were incubated with the corresponding antibodies at 4 °C overnight. The antibody information is listed in Supplementary Table 3.

### Statistical analysis

GraphPad Prism Version 8.0 was used for statistical analysis. All data are expressed as the mean ± standard deviation (SD). Student’s *t* test or Welch’s *t* test was used to determine significant differences among the groups. One-way ANOVA was used for multiple tests. Comparisons among treatment groups were performed with two-way ANOVA. A *P* value less than 0.05 was considered statistically significant.

## Results

### TGF-β2 induces more pericyte infiltration and subretinal fibrosis in mice with laser-induced CNV

**Fig. 1:**
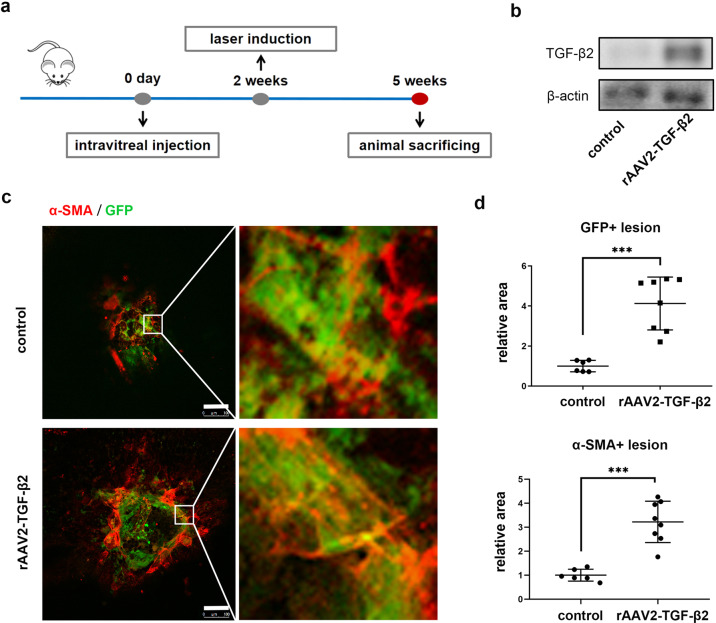
TGF-β2 enhanced pericyte infiltration and transition to myofibroblast in laser-induced Col1α1-GFP transgenic mice. **a** Schematic diagram of intravitreal injection of rAAV2-TGF-β2 or normal saline and laser photocoagulation in Col1α1-GFP transgenic mice. **b** Western blotting analysis of TGF-β2 in the vitreous humor of mice 2 weeks after intravitreal injection of rAAV2-TGF-β2 or normal saline. **c** Immunofluorescence analysis of RPE-choroid complex flatmounts. Green, GFP; Red, α-SMA. Scale bar, 100 μm. **d** Quantitative measurements of GFP-positive areas and α-SMA-positive areas in Panel **c** (*n* = 6–8 per group). The data are presented as the mean ± SD. Statistical analyses were performed by Welch’s *t* test. ***P < 0.001.

Previously, we reported that GFP-positive pericytes were a significant component of subretinal lesions in a laser-induced CNV mouse model^13^. It would be interesting to know if these GFP-positive pericytes could be activated by TGF-β2 and transformed into myofibroblasts, resulting in subretinal fibrosis. To verify this hypothesis, rAAV2-TGF-β2 was injected intravitreally to increase intraocular TGF-β2 levels in mice; 2 weeks later, laser photocoagulation was performed (Fig. 1a). Western blotting first demonstrated an increase in the protein level of TGF-β2 in vitreous humor 2 weeks after intravitreal injection of rAAV2-TGF-β2 (Fig. 1b). Compared with those in the control group, the areas of immunostaining for the myofibroblast markerα-SMA and GFP-positive pericytes were increased in laser spots in rAAV2-TGF-β2-treated Col1α1-GFP transgenic mice (Fig. 1c, d), which was approximately 4.1-fold (GFP) and 3.2-fold (α-SMA) of that of the normal control. Moreover, obvious colocalization of GFP and α-SMA was detected in laser-treated mice, confirming that PMT was involved in subretinal fibrosis. These data demonstrated that TGF-β2 enhances GFP-positive pericyte infiltration and the transition to myofibroblasts in a laser-induced CNV model.

Previous studies reported that TGF-β2 can also trigger the transition to myofibroblasts or induce the expression of type I collagen in other cell types^11^. Thus, in addition to GFP-positive pericytes, other cells might also be involved in myofibroblast transition after TGF-β2 stimulation within CNV lesions following intravitreal injection of rAAV2-TGF-β2. To characterize the specific cell types involved in the myofibroblast transition, we performed coimmunostaining with different antibodies and GFP on cryosections of subretinal lesions in mice treated with intravitreal injection of rAAV2-TGF-β2 and laser photocoagulation, as shown in Fig. 1a. The data showed that GFP-positive cells at the lesion site colocalized with PDGFRβ, indicating their pericyte origins (Supplementary Fig. 1a). However, other markers, including BEST1 (a specific marker of RPE cells), CD31 (a specific marker of endothelial cells) and GS (a specific marker of Müller cells), were not colocalized with GFP-positive cells (Supplementary Fig. 1a). Pericytes in the inner blood–retinal barrier might be induced by the increase in TGF-β2 in the vitreous humor and migrate to the subretinal space to participate in the formation of CNV lesions. To determine the involvement of pericytes, we performed immunostaining of PDGFRβ on both retinal cryosections and flatmounts (Supplementary Fig. 1b, c) from mice with laser-induced CNV. The data showed that no PDGFRβ-positive cells were observed in the outer retina and subretinal space in retinal cryosections (Supplementary Fig. 1b). Immunostaining of retinal flatmounts further confirmed that PDGFRβ-positive cells were localized in the inner blood–retinal barrier and did not migrate to the outer retina (Supplementary Fig. 1c). These results showed that retinal pericytes were not involved in CNV lesions in mice with laser-induced CNV, and GFP-positive pericytes in CNV lesions originated from choroidal pericytes treated with rAAV2-TGF-β2.

To rule out the possible effects of the viral vector on PMT, C57BL/6J mice were injected intravitreally with the rAAV2 vector or normal saline, followed by laser photocoagulation. The results showed that there were no significant differences in pericytes or myofibroblasts between these two groups (Supplementary Fig. 1d), indicating that the viral vector had no effect on PMT.

Overall, TGF-β2 enhances choroidal pericyte infiltration and its involvement in subretinal fibrosis in a laser-induced CNV model.

### TGF-β2 promotes pericyte differentiation, proliferation and migration in vitro

**Fig. 2:**
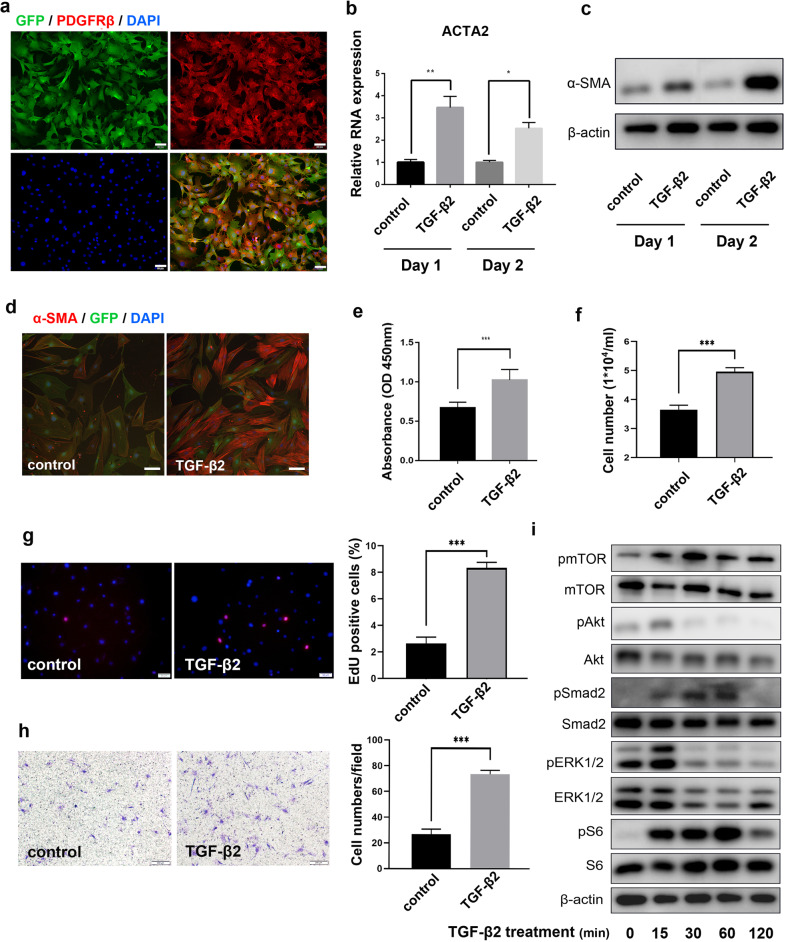
TGF-β2 promoted pericyte differentiation and proliferation in vitro. **a** Primary pericytes isolated from Col1α1-GFP transgenic mice were immunostained with PDGFRβ and DAPI. Green, GFP; Red, PDGFRβ; Blue, DAPI. Scale bar, 50 μm. **b** The mRNA expression of ACTA2 in primary pericytes with or without TGF-β2 treatment. **c** The protein expression of α-SMA in primary pericytes with or without TGF-β2 treatment. **d** Immunofluorescence analysis of α-SMA in mouse primary pericytes 2 days after TGF-β2 treatment. Green, GFP; Red, α-SMA; Blue, DAPI. Scale bar, 50 μm. **e** CCK8 analysis of primary pericytes treated with or without TGF-β2. **f** Cell counting measurements. The number of viable cells was measured by a hemocytometer. **g** EdU assay to assess cell proliferation. Cell nuclei with high DNA replication activity (EdU-positive cells) were stained red. Scale bar, 50 μm. **h** Transwell migration assay. The migrated cells were counted in three fields to determine the average number of migrated cells per field. Scale bar, 200 μm. **i** The prote**i**n expression levels of mTOR, Akt, Smad2, ERK and S6 were measured by Western blotting in primary pericytes treated with TGF-β2 for different times. The data are presented as the mean ± SD, and statistical analyses were performed by Welch’s *t* test. ns, nonsignificant difference. *P < 0.05, **P < 0.01, ***P < 0.001.

To further investigate the effect of TGF-β2 on choroidal pericytes, we isolated and cultured primary pericytes. As shown in Fig. 2a, all GFP-positive cells expressed PDGFRβ (a specific marker of pericytes), indicating that these GFP-positive primary cells were pericytes. Immunostaining for other markers, including BEST1, CD31 and GFAP (a specific marker of macroglia), was negative (Supplementary Fig. 2a), further confirming the purity of these primary pericytes. After TGF-β2 treatment, the mRNA expression of ACTA2 (Fig. 2b) and the protein expression of α-SMA (Fig. 2c) were upregulated significantly. Notably, 2 days after TGF-β2 treatment, the mRNA expression of α-SMA was approximately 2.56-fold (*n* = 3) of that in the control (Fig. 2b). To further confirm the effect of TGF-β2, immunofluorescence analysis of α-SMA was performed, and the results showed that TGF-β2 treatment enhanced the differentiation of pericytes to myofibroblasts (Fig. 2d). To evaluate the effect of TGF-β2 on primary choroidal pericyte proliferation, CCK8 assays, cell counting measurements and EdU staining were performed. One day after TGF-β2 treatment, the OD450 values (Fig. 2e) of the CCK8 assay and cell counts (Fig. 2f) were increased significantly. Additionally, as shown in Fig. 2g, TGF-β2 significantly increased the number of EdU-positive cells. These results suggested that TGF-β2 treatment could increase the proliferation of primary choroidal pericytes. In addition to promoting cell differentiation and proliferation, we also examined the migration of primary pericytes treated with or without TGF-β2. As measured with a Transwell migration assay, the migration of pericytes after treatment with TGF-β2 for 24 h was significantly higher than that in the control group (Fig. 2h), suggesting that TGF-β2 promotes the migration of primary choroidal pericytes. Unexpectedly, in the wound healing experiment, TGF-β2 showed no obvious effect on gap sizes at different time points, but a distinct spindle-like morphology was observed in TGF-β2-treated cells (Supplementary Fig. 2b), which also indicated that primary choroidal pericytes underwent PMT after TGF-β2 induction.

In addition, to examine the changes in the signaling pathways induced by TGF-β2, the protein expression levels of mTOR, Akt, Smad2, ERK1/2, and S6 in primary pericytes treated with or without TGF-β2 were measured by Western blotting. As shown in Fig. 2i, the phosphorylation of these proteins was increased 15 min after TGF-β2 treatment, but the patterns differed; the phosphorylation of Akt and ERK1/2 decreased after 15 min, while the phosphorylation of Smad2, mTOR and S6 persisted for more than 1 h.

### SMAD2/3 and Akt/mTOR signaling contribute to TGF-β2-induced PMT in vitro

**Fig. 3:**
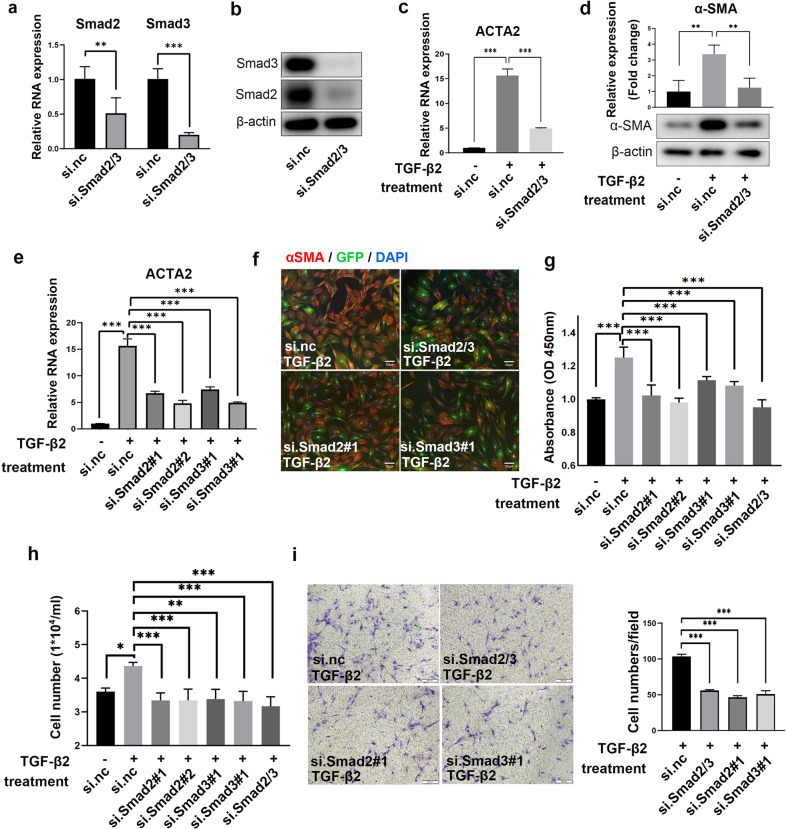
TGF-β2-induced pericyte-myofibroblast transition and proliferation was Smad2/3 dependent. **a, b** Changes in the mRNA (**a**) and protein (**b**) levels of Smad2 and Smad3 in primary pericytes treated with siRNA (si.Smad2/3). Control small interfering RNA (si.nc) was used as a control. **c** The mRNA expression of ACTA2 in TGF-β2-treated primary pericytes with or without si.Smad2/3 for 24 h (*n* = 3). **d** The protein expression of α-SMA in TGF-β2-treated primary pericytes with or without si.Smad2/3 for 48 h (*n* = 3). **e** The mRNA expression of ACTA2 in TGF-β2-treated primary pericytes with or without si.Smad2 or si.Smad3 (*n* = 3). **f** Immunofluorescence analysis of α-SMA in TGF-β2-treated primary pericytes with or without Smad2/3 siRNA treatment. Green, GFP; Red, α-SMA; Blue DAPI. Scale bar, 50 μm. **g** CCK8 analysis of TGF-β2-treated primary pericytes with or without Smad2/3 siRNA treatments. **h** Cell counting measurements. The number of viable cells was measured by a hemocytometer. **i** Transwell migration assay. The migrated cells were counted in three fields to determine the average number of migrated cells per field. Scale bar, 200 μm. The data are presented as the mean ± SD, and statistical analyses were performed by Welch’s *t* test or one-way ANOVA. **P < 0.01, ***P < 0.001.

Considering that Smad2/3 is the canonical downstream signaling pathway of TGF-β, we knocked down Smad2/3 in primary pericytes to determine whether Smad2/3 was necessary for the transition to myofibroblasts. Both the mRNA and protein levels of Smad2/3 were significantly decreased after transfection with si.Smad2/3 (Fig. 3a, b). As expected, knockdown of Smad2/3 reduced the mRNA (ACTA2) and protein expression of α-SMA in TGF-β2-treated primary pericytes (Fig. 3c, d). To determine which molecule plays a key role in PMT, Smad2 and Smad3 were knocked down separately (Supplementary Fig. 3a, b), and the mRNA level of ACTA2 was decreased significantly by si.Smad2 or si.Smad3 (Fig. 3e). Immunostaining for α-SMA also confirmed that the inhibition of Smad2/3 alone or in combination decreased PMT in TGF-β2-treated primary pericytes (Fig. 3f). The OD450 values in the CCK8 assay (Fig. 3g) and cell counts (Fig. 3h) were decreased in TGF-β2-treated primary pericytes after the knockdown of Smad2, Smad3, or both; EdU-positive cells were also reduced by si.Smad2/3 (Supplementary Fig. 3c), indicating that Smad2/3 mediated cell proliferation in addition to PMT. Moreover, as shown in Fig. 3i, Smad2/3 inhibition also suppressed the TGF-β2-induced migration of primary pericytes.

**Fig. 4:**
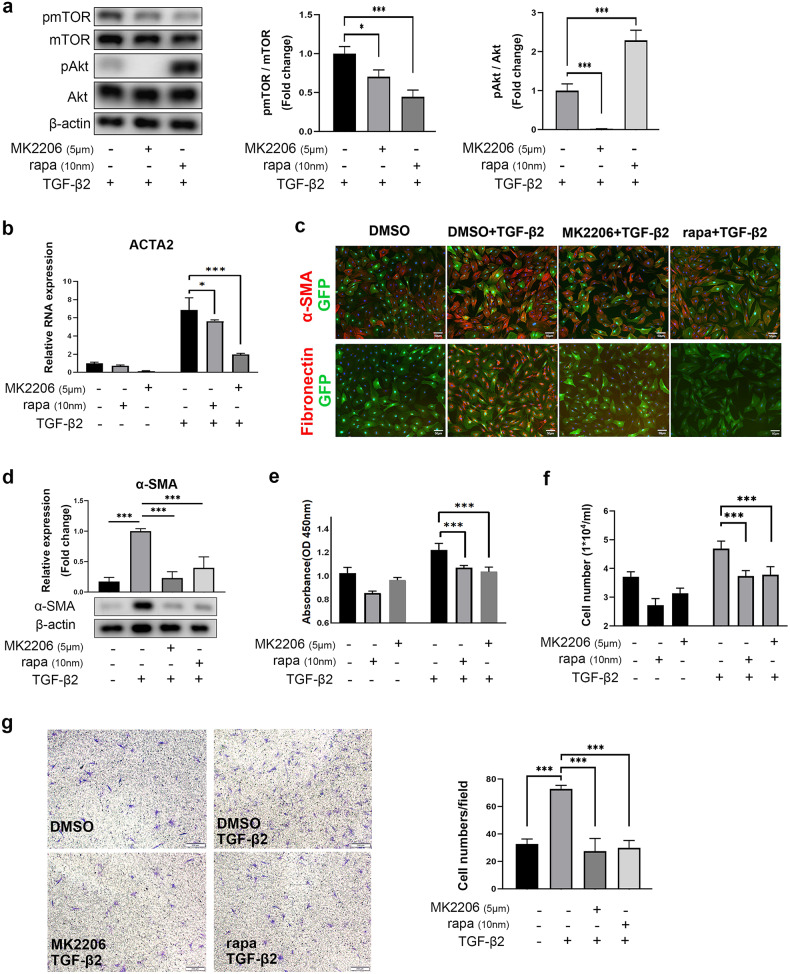
Akt/mTOR signaling is necessary for PMT, proliferation and migration in TGF-β2-treated pericytes. **a** The protein expression of mTOR and Akt in TGF-β2-treated primary pericytes treated with MK2206 (Akt inhibitor) or rapa (rapamycin, mTOR inhibitor). **b** The mRNA expression of ACTA2 in TGF-β2-treated primary pericytes treated with MK2206 or rapa (*n* = 3). **c** Immunofluorescence analysis of α-SMA and fibronectin in TGF-β2-treated primary pericytes treated with MK2206 or rapa. Green, GFP; Red, α-SMA or fibronectin. Scale bar, 50 μm. **d** The protein expression of α-SMA in TGF-β2-treated primary pericytes treated with MK2206 or rapa (*n* = 3). **e** CCK8 analysis of TGF-β2-treated primary pericytes treated with MK2206 or rapa. **f** Cell counting measurements. The number of viable cells was measured by a hemocytometer. **g** Transwell migration assay. The migrated cells were counted in three fields to determine the average number of migrated cells per field. Scale bar, 200 μm. The data are presented as the mean ± SD, and statistical analyses were performed by one-way ANOVA or two-way ANOVA. *P < 0.05, ***P < 0.001.

In addition to the TGF-β2/Smad2/3 signaling pathway, the Akt/mTOR pathway (Fig. 2i) was also examined in TGF-β2-treated primary pericytes. The Akt inhibitor MK2206 downregulated the phosphorylation of mTOR, while the mTOR inhibitor rapamycin activated Akt signaling, which might be due to a negative feedback response to mTOR inhibition, indicating that mTOR is a downstream target of Akt (Fig. 4a). After MK2206 or rapamycin treatment, the TGF-β2-induced increases in mRNA (ACTA2) and protein (α-SMA) levels were attenuated (Fig. 4b, d). Immunofluorescence analysis of α-SMA and fibronectin also confirmed the effect of MK2206 or rapamycin, and the immunostaining of α-SMA and fibronectin was obviously decreased in TGF-β2-induced primary pericytes treated with MK2206 or rapamycin (Fig. 4c). In addition, cell proliferation was also significantly decreased in TGF-β2-induced primary pericytes treated with MK2206 or rapamycin (Fig. 4e, f, Supplementary Fig. 4a). Interestingly, inhibiting Akt/mTOR significantly suppressed migration in TGF-β2-treated pericytes, which was detected both in Transwell migration assays and wound healing experiments (Fig. 4g, Supplementary Fig. 4b). Moreover, the spindle-like morphology of pericytes induced by TGF-β2 was largely affected by MK2206 or rapamycin (Supplementary Fig. 4b). Briefly, TGF-β2-treated pericytes exhibited a short spindle or oat-like morphology when treated with MK2206 but showed a more elongated morphology when treated with rapamycin.

Inhibiting of ERK1/2 phosphorylation with SCH772984 (ERKi, Selleck Chemicals) decreased cell proliferation but had no significant effect on α-SMA protein expression (Supplementary Fig. 5a–c).

### Smad2/3 synergizes with Akt/mTOR to mediate PMT and cell proliferation in primary pericytes

**Fig. 5:**
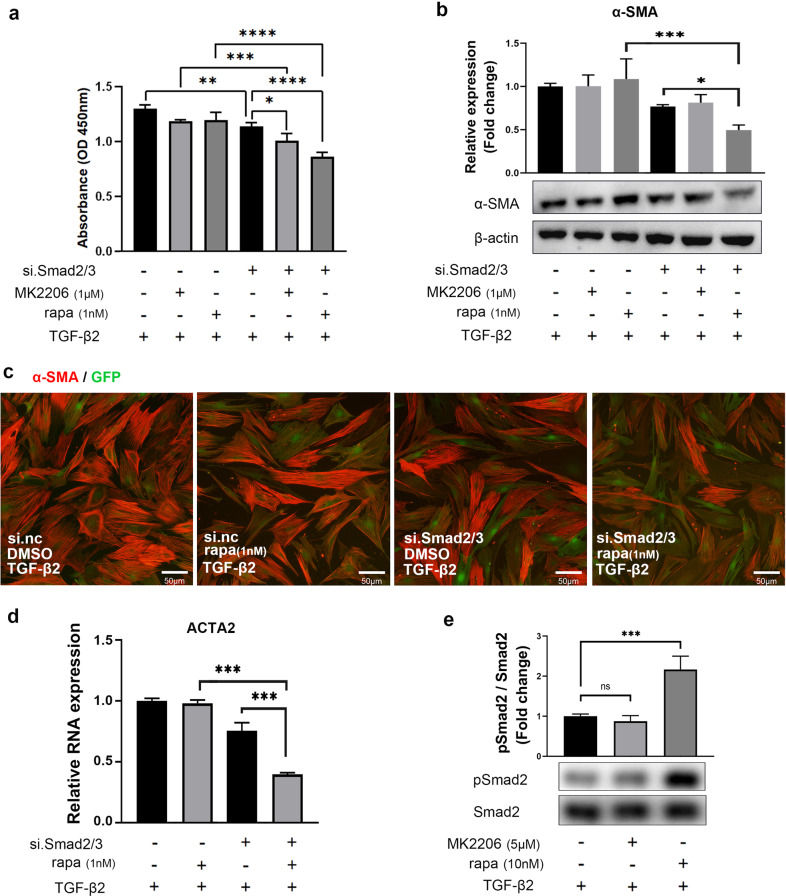
The synergistic effect of Smad2/3 and mTOR inhibition suppressed the proliferation and differentiation of primary pericytes. **a** CCK8 analysis of primary pericytes in the different groups. **b** Changes in α-SMA protein expression in TGF-β2-treated primary pericytes treated with si.Smad2/3, MK2206 or rapa. **c** Immunofluorescence analysis of α-SMA protein expression in TGF-β2-treated primary pericytes treated with si.Smad2/3, MK2206 or rapa. Green, GFP; Red, α-SMA; scale bar, 50 μm. **d** Changes in mRNA expression of ACTA2 in TGF-β2-treated primary pericytes treated with si.Smad2/3 or rapa (*n* = 3). **e** The mTOR inhibitor (rapa) increased the phosphorylation of Smad2 in TGF-β2-treated primary pericytes. The data are presented as the mean ± SD, and statistical analyses were performed by one-way ANOVA or two-way ANOVA. ns, nonsignificant difference. *P < 0.05, **P < 0.01, ***P < 0.001.

Since the Smad2/3 and Akt/mTOR signaling pathways are both essential for PMT (Figs. 3c–f and b–d) and cell proliferation (Figs. 3g, h and e, f) in TGF-β2 induced primary pericytes, the synergistic effect of the Smad2/3 and Akt/mTOR signaling pathways in the presence of low-dose inhibitors was explored to avoid the potential side effects of excessive inhibition caused by high-dose inhibitors. Primary pericytes were transfected with siRNA for Smad2/3 in low-dose RFect transfection reagent (0.166%), followed by treatment with low doses of the inhibitors MK2206 (1 μM) or rapamycin (1 nM) for 2 h, and then TGF-β2-induced PMT or cell proliferation was assessed. As shown in Fig. 5a, the combined inhibition of Smad2/3 and Akt/mTOR greatly decreased cell proliferation compared to single inhibition. The simultaneous inhibition of Smad2/3 and mTOR induced significant decreases the protein expression of α-SMA compared to single inhibition (Fig. 5b). The combined effect was also validated by decreased immunostaining of α-SMA (Fig. 5c) and reduced mRNA expression (Fig. 5d). These findings suggest that combined inhibition of the Smad2/3 and mTOR signaling pathways with low-dose inhibitors can achieve significant antifibrotic effects in pericytes. Unexpectedly, combined mild inhibition of Smad2/3 and Akt showed no obvious effect on attenuating α-SMA expression, possibly because a low dose of MK2206 was not sufficient to inhibit Akt. Interestingly, rapamycin upregulated the phosphorylation of Smad2 in primary pericytes (Fig. 5e), indicating crosstalk between Smad2/3 and mTOR, which deserves further exploration.

### Akt/mTOR is required for PMT and contributes to subretinal fibrosis in vivo

The above data showed that Smad2/3 synergized with Akt/mTOR and could promote cell proliferation (Fig. 5a) and PMT (Fig. 5b–d) in TGF-β2-induced primary pericytes; thus, combined inhibition of Smad2/3 and mTOR might be a novel strategy to treat subretinal fibrosis. To further explore the mechanism of subretinal fibrosis, we examined the involvement of these signaling pathways in laser-induced CNV mouse models. The phosphorylation levels of Smad2 and S6 were increased significantly in the RPE-choroid complex of CNV models compared with the control, while there was no significant change in ERK1/2 between these two groups (Supplementary Fig. 6a). Moreover, phosphorylated S6 was examined and colocalized with GFP-positive pericytes in CNV (Supplementary Fig. 6b). Considering that phosphorylated S6 is generally a good indicator of ribosomal protein S6 kinase (S6K) activity, which is indirectly downstream of mTOR^26^, these results suggested that Akt/mTOR activation was involved in PMT in laser-induced CNV, which was consistent with the in vitro results.

**Fig. 6:**
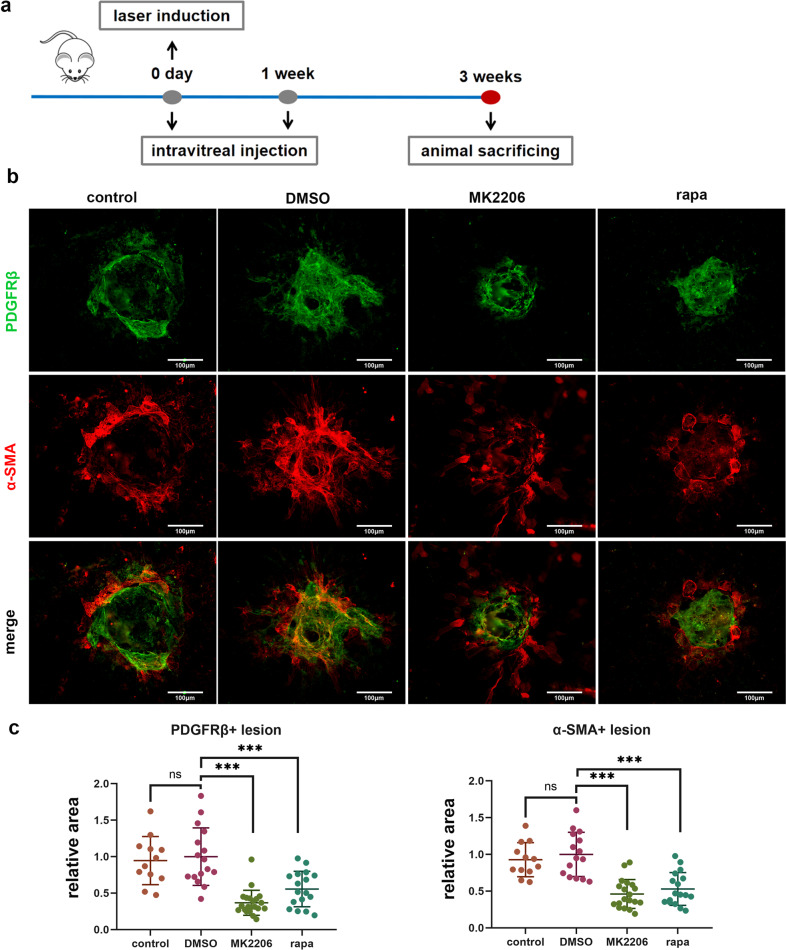
Rapamycin suppressed pericyte infiltration and subretinal fibrosis in vivo. **a** Schematic diagram of the establishment of the laser-induced CNV mouse model and intravitreal injection. **b** Immunofluorescence analysis of PDGFRβ and α-SMA in RPE-choroid complex flatmounts. Green, PDGFRβ; Red, α-SMA. Scale bar, 100 μm. **c** Quantitative measurements of PDGFRβ-positive areas and α-SMA-positive areas (*n* = 11–19 per group) in **b**. The data are presented as the mean ± SD. Statistical analyses were performed by one-way ANOVA. ns, nonsignificant difference. ***P < 0.001.

Based on these findings, we hypothesized that inhibition of the Akt/mTOR pathway might inhibit or slow PMT and thus the development of subretinal fibrosis caused by choroidal pericytes in laser-induced CNV. To determine the optimal treatment, two regimens of MK2206 or rapamycin administration were used for early intervention (Fig. 6a) and late intervention (Supplementary Fig. 7a). There were no significant differences in PDGFRβ-positive lesion sizes or α-SMA-positive lesion sizes between vehicle (DMSO) and untreated control eyes in response to either regimen (Fig. 6b, Supplementary Fig. 7b). The lesion sizes, which were indicated by the PDGFRβ-positive area or α-SMA-positive area, were decreased significantly by early intervention with MK2206 or rapamycin compared with those in the vehicle (DMSO) group (Fig. 6b, c). Late intervention showed no significant effects on suppressing lesion sizes (Supplementary Fig. 7b, c). Thus, early treatment with Akt/mTOR signaling pathway inhibitors is of great importance for treating subretinal fibrosis.

## Discussion

Subretinal fibrosis represents the end stage of neovascular AMD and develops progressively despite successful anti-VEGF therapy^2,7,12^. Multiple cell types and cytokines contribute to the progression of CNV to fibrosis. Current evidence suggests that RPE cells, endothelial cells, Müller cells, and macrophages can differentiate into myofibroblasts, which are the main cell type that induces subretinal fibrosis and produces ECM components^12^. However, the mechanism of subretinal fibrosis remains largely unexplored, and effective treatments are lacking. Based on our previous study^13^, we found that choroidal pericytes, after being stimulated with TGF-β2, underwent increased proliferation and transition to myofibroblasts, which was dependent on the activation of the Smad2/3 and Akt/mTOR signaling pathways. In addition, early inhibition of the Akt/mTOR signaling pathway was effective in alleviating subretinal fibrosis.

Pericytes are vascular mural cells that are embedded within the vascular basement membrane of blood microvessels, where they make specific focal contacts with the endothelium^27^. As a heterogeneous population, pericytes vary greatly in morphology and molecular biology in different tissues^28^. Pericytes have been reported to have various functions, such as capillary constriction, the regulation of blood flow, and angiogenesis^27^ and may act as progenitors involved in tissue regeneration^29^. The role of pericytes as myofibroblast precursors has also been suggested by studies of fibrogenesis in the liver, kidney, lung, and systemic sclerosis^22,28,30^ However, the detailed mechanisms of PMT in subretinal fibrosis have not been previously reported in choroids, which caught our attention based on our previous study^13^. In the present study, primary pericytes were isolated from choroids and were used, for the first time, to study the underlying mechanisms for PMT and fibrosis, which provided potential therapeutic targets for suppressing subretinal fibrosis.

TGF-β is a central profibrotic mediator and a master regulator that induces mesenchymal transition in a variety of cells^21^. TGF-β is expressed in surgically removed CNV tissue^31^. In mice with subretinal fibrosis, TGF-β is also strongly upregulated, and systemic administration of TGF-β-neutralizing antibodies ultimately reduces subretinal fibrosis^32^. In the present study, we found that TGF-β2 increased the infiltration of choroidal pericytes in subretinal fibrosis, as well as the differentiation, proliferation and migration of primary choroidal pericytes in vitro. Interestingly, although Transwell migration assays showed that TGF-β2 significantly promoted the migration of pericytes (Fig. 2h), the wound healing assays showed no differences in the gap sizes between the control group and the TGF-β2 group at different time points (Supplementary Fig. 2b). The reason that no significant differences was detected in the wound healing assay might be that primary choroidal pericytes at the edge of the scratch were more responsive to mechanical stimulation by the scratch, which merits further exploration. TGF-β ligands bind to the receptor TGF-βR2, which phosphorylates TGF-βR1 and ultimately increases gene expression and promotes ECM formation. During this process, multiple downstream regulators are activated, such as Smad2/3 signaling, as well as non-Smad pathways, such as mitogen-activated protein kinase (MAPK) pathways, Rho-like GTPase signaling pathways, and phosphatidylinositol-3-kinase (PI3K)/Akt/mTOR pathways^21,33^. Although Smad2 and Smad3 are highly homologous and share some overlapping activities, they have distinct functions and are regulated differentially^34,35^. In the present study, both Smad2 and Smad3 were important for TGF-β2-mediated proliferation, differentiation and migration of choroidal pericytes (Fig. 3c–i). mTOR is a serine/threonine protein kinase in the PI3K-related kinase (PIKK) family that forms the catalytic subunit of two distinct protein complexes known as mTOR complex 1 (mTORC1) and 2 (mTORC2). Rapamycin inhibits mTORC1 activity allosterically, while mTORC2 exhibits short-term rapamycin insensitivity. mTORC1 activation is regulated by growth factors, nutrients, energy status, and stress. In our study, mTOR activity decreased but remained at a certain level after Akt inhibition (Fig. 4a), suggesting that mTOR activity in choroidal pericytes might be partially regulated by Akt. This finding was consistent with previous studies that showed that multiple upstream signals were involved in regulating mTORC1 activity, such as amino acids and WNT signaling^36,37^. Interestingly, in agreement with a previous study^38^, we showed negative feedback for the first time in choroidal pericytes and that Akt phosphorylation was increased upon mTORC1 inhibition by rapamycin (Fig. 4a).

The Smad2/3 pathway integrates intracellular signals through crosstalk with other signaling pathways, playing important roles in the regulation of various biological responses^39^. A highlight of this study is that Smad2/3 and mTOR acted synergistically to induce choroidal pericyte proliferation (Fig. 5a) and the transition to myofibroblasts (Fig. 5b–d). Moreover, consistent with a previous study^40^, we revealed that mTOR inhibition significantly upregulated the phosphorylation of Smad2, which could counteract the therapeutic effect of rapamycin on PMT. This finding might partially explain the significant effect of the coadministration of mTOR and Smad2/3 inhibitors on PMT. Further study is needed to investigate the mechanism of this crosstalk between Smad2/3 and AKT/mTOR in pericytes.

Several studies have reported the effects of various Akt/mTOR inhibitors on suppressing laser-induced CNV. For example, intraperitoneal rapamycin (4 mg/kg/day) reduced the area of laser-induced CNV on Day 14 in mice, but statistically significant differences were not observed, and the mechanism was not explored^41^. Another study showed that oral or intravitreal administration of GSK2126458, a highly selective inhibitor of the class I PI3K/mTOR pathway, significantly reduced the leakage and areas of CNV lesions on Day 14 and 16^42^. Recently, rAAV-delivered mTOR-inhibiting short hairpin RNA (rAAV-mTOR shRNA), which blocks both mTORC1 and mTORC2, was confirmed to suppress laser-induced CNV in mice on Day 14^43^, and mTORC1 was shown to play an important role in CNV in response to systemic rapamycin^44^. In addition, a clinical study of neovascular AMD patients noted that rapamycin decreased the need for anti-VEGF intravitreal injections^45^. Although these studies showed that Akt/mTOR inhibition was safe and effective for controlling neovascularization, the effects of Akt/mTOR inhibition on subretinal fibrosis secondary to CNV remain unclear. Indeed, it has been widely reported that the Akt/mTOR pathway plays a master role in pulmonary fibrosis, and several Akt/mTOR inhibitors in development or in clinical trials have been reported to minimize clinical pulmonary fibrosis^46,47^. Based on these studies and our findings in vitro, we used Akt/mTOR as the target to test whether inhibition of this pathway could suppress pericyte activation and attenuate subretinal fibrosis. By using different regimes, we ultimately revealed that early intervention might achieve better outcomes than late intervention in suppressing PMT (Fig. 6a–c, Supplementary Fig. 5a–c).

In conclusion, the current study demonstrated the involvement of choroidal pericytes in PMT and subretinal fibrosis through the Smad2/3 and Akt/mTOR signaling pathways in response to TGF-β stimulation. This study also presented the first evidence of the therapeutic value of Akt/mTOR inhibitors, especially rapamycin, in controlling subretinal fibrosis and provided a relatively effective treatment regimen for the use of rapamycin in subretinal fibrosis that emphasizes early intervention.

## Supplementary information

Supplementary data

## Data Availability

The data that support the findings of this study are available from the corresponding author upon reasonable request. Supplementary information is available for this paper.
