# Author Correction: TGF-β promotes pericyte–myofibroblast transition in subretinal fibrosis through the Smad2/3 and Akt/mTOR pathways

**DOI:** 10.1038/s12276-026-01786-0

**Published:** 2026-06-29

**Authors:** Zhenzhen Zhao, Yumeng Zhang, Chaoyang Zhang, Jingting Zhang, Xueting Luo, Qinghua Qiu, Dawei Luo, Jingfa Zhang

**Affiliations:** 1 Department of Ophthalmology, Shanghai General Hospital (Shanghai First People’s Hospital), Shanghai Jiao Tong University, School of Medicine, Shanghai, China; 2 National Clinical Research Center for Eye Diseases, Shanghai Key Laboratory of Ocular Fundus Diseases, Shanghai Engineering Center for Visual Science and Photomedicine, Shanghai Engineering Center for Precise Diagnosis and Treatment of Eye Diseases, Shanghai, China; 3 Department of Ophthalmology, Shigatse People’s Hospital, Xizang, China

**Keywords:** Growth factor signalling, Transforming growth factor beta

Correction to: *Experimental & Molecular Medicine* 10.1038/s12276-022-00778-0, published online 27 May 2022

In Fig. 4c, the fibronectin staining image for the rapamycin‑treated group (bottom‑right panel) was inadvertently replaced with a duplicate of the DMSO control group image. The correct original image for the rapamycin‑treated group has been included in the corrected version of Fig. 4c. This correction is for representative illustration only and does not affect any data analyses, results, interpretations, or conclusions reported in the article.

Incorrect figure
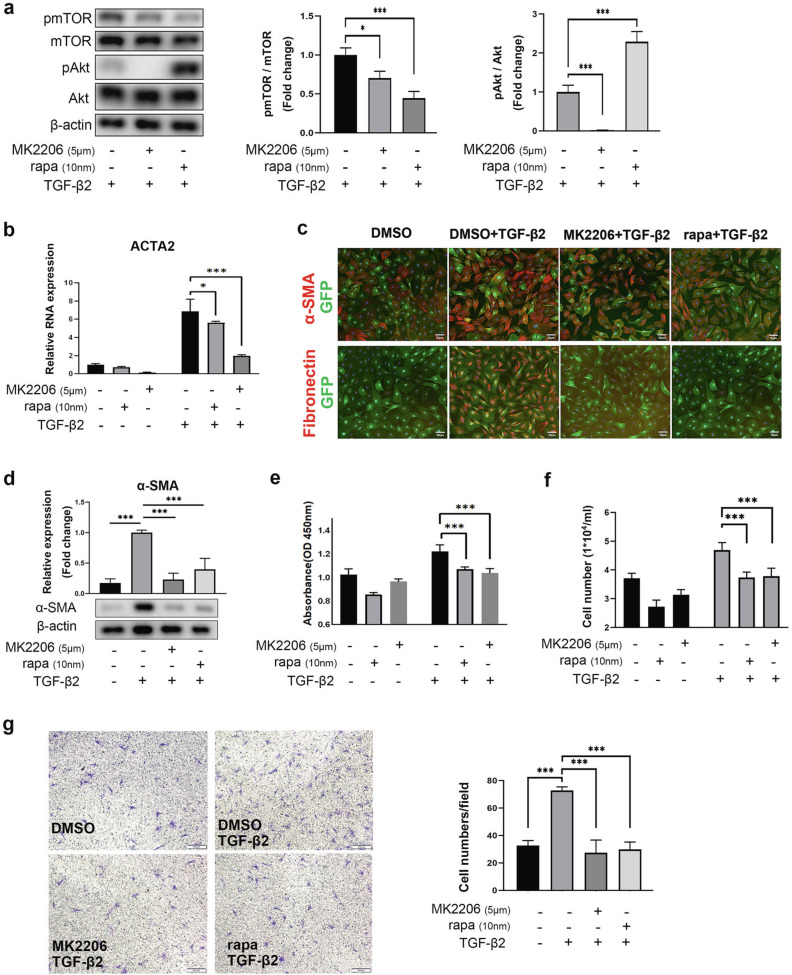


Correct figure
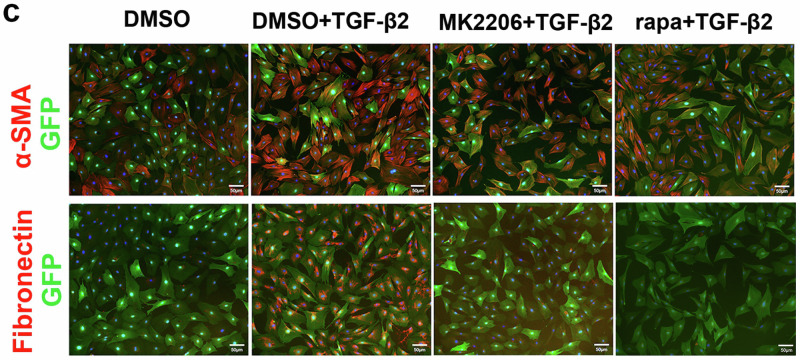


The original article has been corrected.

