# Supplementary material for: TGF-β promotes pericyte-myofibroblast transition in subretinal fibrosis through the Smad2/3 and Akt/mTOR pathways
Source: Exp Mol Med. 2022 May 27;54(5):673–84. doi: 10.1038/s12276-022-00778-0 (PMC9166792; doi:10.1038/s12276-022-00778-0)
Supplement: Supplementary file 1 — Supplementary data [file 12276_2022_778_MOESM1_ESM.pdf]

Supplementary data

Supplementary Table1. Information for siRNAs

siRNA	Sense (5'-3')	Antisense (5'-3')
si.Smad2#1	GAAUUGAGCCACAGAGUAA	UUACUCUGUGGCUCAAUUC
si.Smad2#2	GGAUUGAACUUCAUCUGAA	UUCAGAUGAAGUUCAAUCC
si.Smad3#1	GCUUGGUGAAGAAGCUCAA	UUGAGCUUCUUCACCAAGC
si.Smad3#2	CCAGAGCAAUAUUCCAGAA	UUCUGGAAUAUUGCUCUGG
si.nc	UUCUCCGAACGUGUCACGU	ACGUGACACGUUCGGAGAA

Supplementary Table2. Mouse primers information for qPCR

Gene	Sense (5'-3')	Antisense (5'-3')
GAPDH	CAAGGTCATCCATGACAACTTTG	GTCCACCACCCTGTTGCTGTAG
ACTA2	CTGACAGAGGCACCACTGAA	CATCTCCAGAGTCCAGCACA
Smad2	ATCTTGCCATTCACTCCGCC	TCTGAGTGGTGATGGCTTTCTC
Smad3	CACGCAGAACGTGAACACC	GGCAGTAGATAACGTGAGGGA

Supplementary Table3. Information for antibodies

Name	Company	Catalog No.	Dilution
β-actin	Proteintech	20536-1-AP	1:1,000 (WB)
GAPDH	Proteintech	60004-1-Ig	1:1,000 (WB)
p-Smad2 (Ser465/467)	Cell Signaling	3108	1:1,000 (WB)
Smad2	Cell Signaling	5339	1:1,000 (WB)
p-ERK1/2(Thr202/Tyr204)	Cell Signaling	4370	1:1,000 (WB)
ERK1/2	Cell Signaling	4695	1:1,000 (WB)
p-Smad3 (Ser423/425)	Cell Signaling	9513	1:1,000 (WB)
Smad3	Cell Signaling	9520	1:1,000 (WB)
p-S6 Ribosomal Protein (Ser235/236)	Cell Signaling	2211	1:1,000 (WB, IF)
S6 Ribosomal Protein	Cell Signaling	2217	1:1,000 (WB)
p-Akt (Ser473)	Cell Signaling	9271	1:1,000 (WB)
Akt Antibody	Cell Signaling	9272	1:1,000 (WB)
p-mTOR (Ser2448)	Cell Signaling	2971	1:500 (WB)
mTOR	Cell Signaling	2972	1:1,000 (WB)
α-SMA	Cell Signaling	14968S	1:1,000 (WB)
Fibronectin	Abcam	ab2413	1:500 (IF)
TGF-β2	Abcam	ab113670	1:1,000 (WB)
GFP	Abcam	ab13970	1:1,000 (IF)
PDGFRβ	Abcam	ab32570	1:100 (IF)
α-SMA	Sigma-Aldrich	C6198	1:1,000 (IF)
GS	Abcam	ab73593	1:1,000 (IF)
CD31	BD Biosciences	BD550274	1:500 (IF)
BEST1	Abcam	ab14927	1：2,000 (IF)
GFAP	Abcam	Ab7260	1:1,000 (IF)

WB: Western blot; IF: immunofluorescence

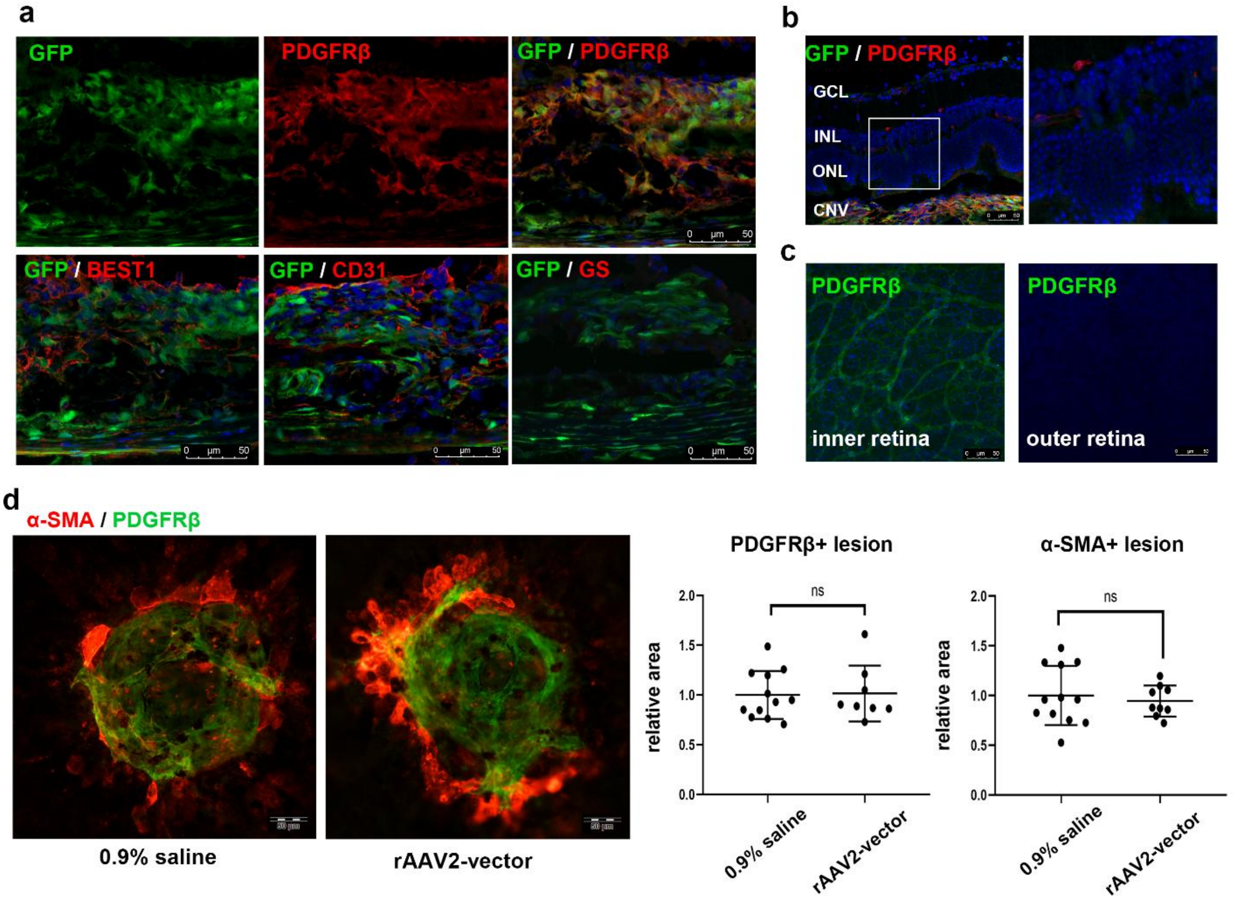

Supplementary Fig.1 Subretinal GFP-positive cells originated from choroidal pericytes. a The co-immunostaining of PDGFRβ, BEST1, CD31, or GS with GFP in CNV lesions 3 weeks after laser-induction in mice following intravitreal rAAV2-TGFβ2 administration. The results showed that GFP was colocalized with PDGFRβ, but not BEST1, CD31, or GS. Scale bar, 50 μm. **b** The co-immunostaining of PDGFRβ with GFP in retinal cryosections in mice after intravitreal rAAV2-TGFβ2 administration. CNV, choroidal neovascularization; GCL, ganglion cell layer; INL, inner nuclear layer; ONL, outer nuclear layer. Scale bar, 50 μm. **c** The immunostaining of PDGFRβ in retinal flatmount both in inner retina and outer retina in mice after intravitreal rAAV2-TGFβ2 administration. Scale bar, 50 μm. **d** Immunofluorescence and quantitation of PDGFRβ and α-SMA in RPE-choroid complex flatmounts. Green, PDGFRβ; Red, α-SMA. Scale bar, 50 μm. Quantitative measurements of PDGFRβ-positive areas and α-SMA-positive areas according to immunostaining (n = 8-12 per group). Data are presented as mean ± SD. Statistics were performed by student's t-test. ns, non-significant difference.

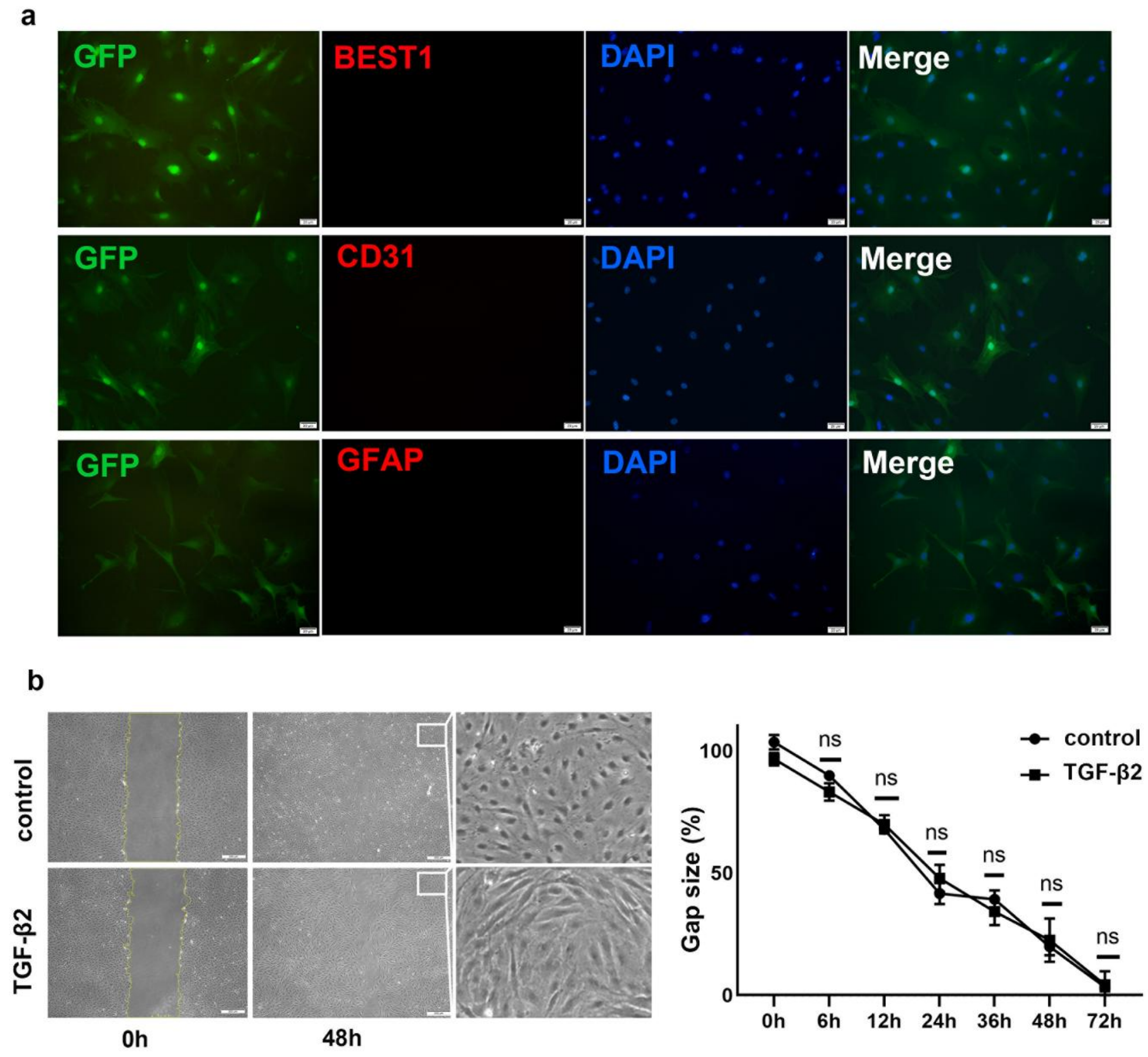

Supplementary Fig.2 Primary Col1α1-GFP cells are pericytes and showed spindle-like morphology after TGF-β2-treatment. a Primary cells were immunostained with antibodies against BEST1 (RPE marker), CD31 (endothelial cell marker), or GFAP (Müller glia and astrocyte marker). All samples were counterstained with DAPI (blue), Scale bar, 20 μm. **b** Wound healing assay of the primary pericytes treated with or without TGF-β2. The enlarged images of the selected rectangle were shown on the right. Scale bar, 200 μm. Data are presented as mean ± SD, statistics were performed by two-way ANOVA. ns, non-significant difference.

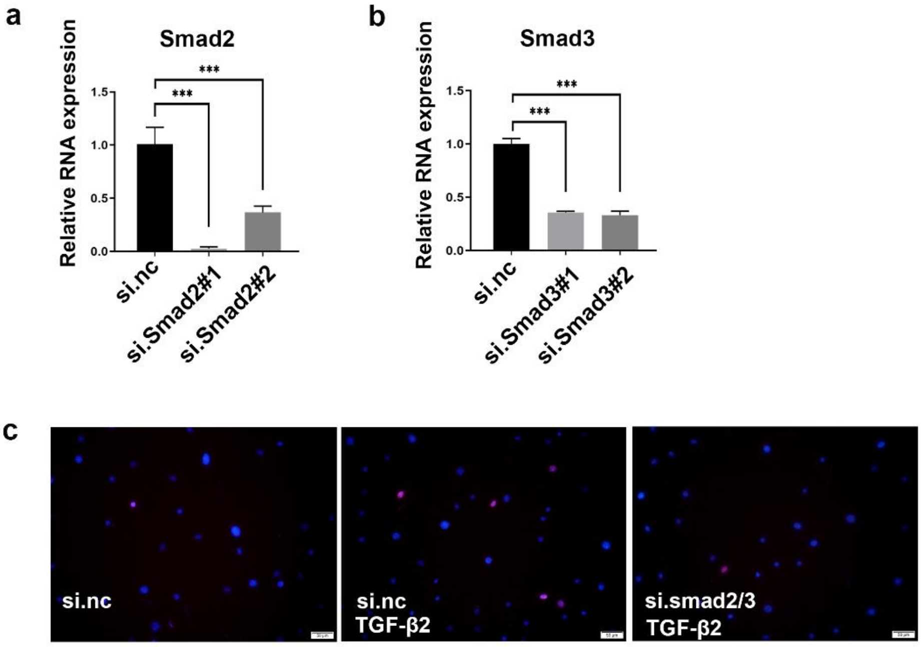

Supplementary Fig.3 Smad2/3 was necessary for the pericyte-myofibroblast transition and the proliferation of primary choroidal pericytes. a, b The mRNA changes of Smad2 and Smad3 in primary pericytes treated with either si.Smad2 (**a**) or si.Smad3 (**b**). **c** EdU assay for cell proliferation. Nuclei of cells with high DNA replication activity (EdU-positive cells) were stained with red. All samples were counterstained with Hoechst (blue), Scale bar, 50 μm. Data are presented as mean ± SD, statistics were performed by one-way ANOVA. ***P < 0.001.

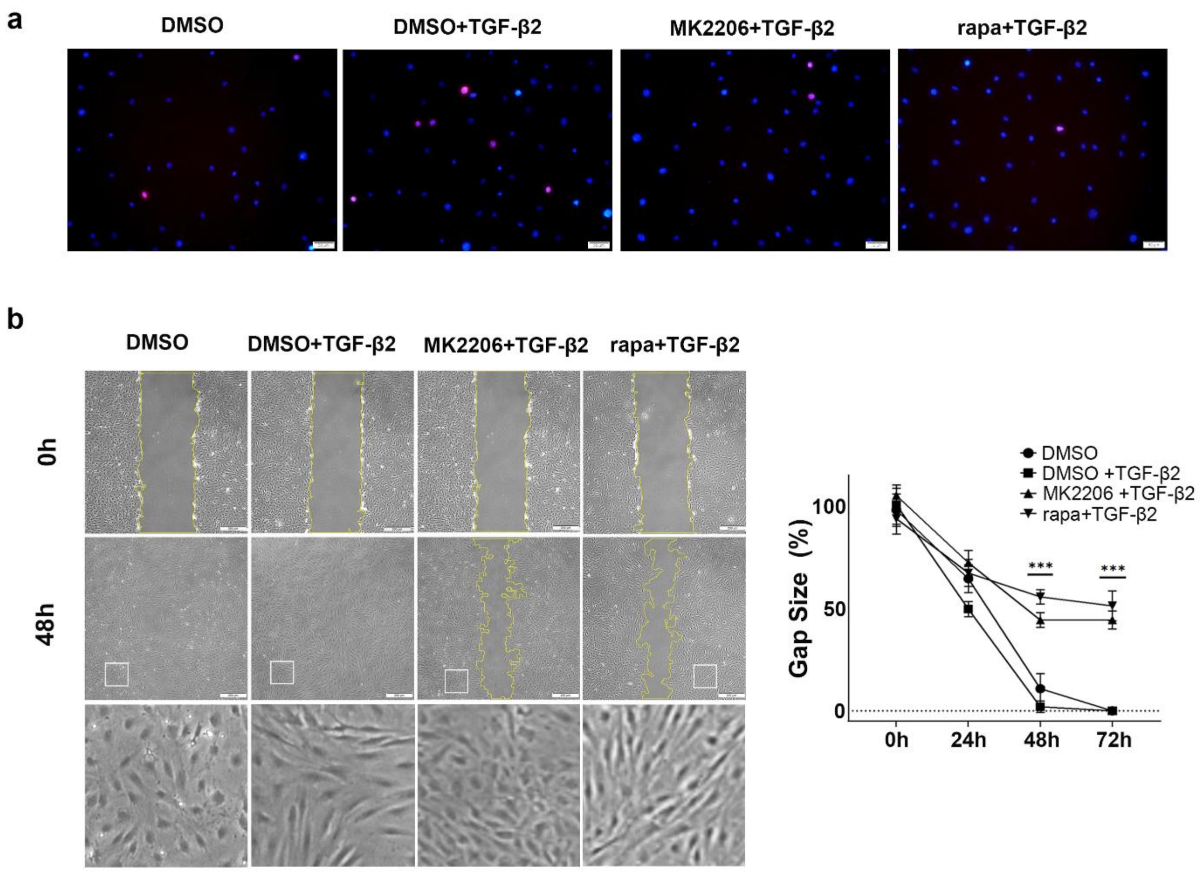

Supplementary Fig.4 Inhibition of Akt/mTOR suppressed proliferation and migration of primary pericytes. a EdU assay for cell proliferation. Nuclei of cells with high DNA replication activity (EdU-positive cells) were stained with red. All samples were counterstained with Hoechst (blue), Scale bar, 50 μm. **b** Wound healing assay of TGF-β2-treated primary pericytes with or without MK2206 or rapa treatments. The enlarged images of the selected rectangle were shown below with obvious morphological changes of cells after treatment with MK2206 or rapa. The diagram illustrated the quantification of gap size calculated as the percentage of the covered area to the initial scratched area (n = 3). Data are presented as mean ± SD, statistics were performed by one-way ANOVA. $^{*}P < 0.05$, $^{***}P < 0.001$.

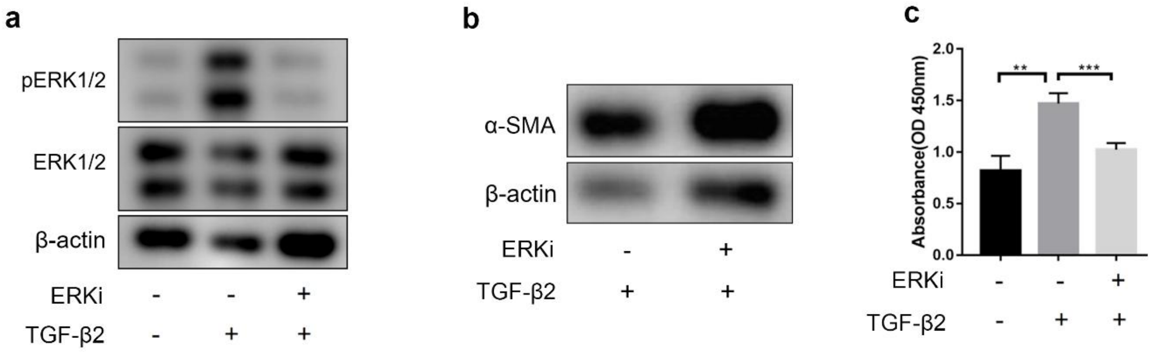

Supplementary Fig.5 Inhibition of ERK1/2 phosphorylation suppressed proliferation of primary pericytes. Primary pericytes were treated with or without ERK1/2 inhibitor (ERKi, SCH772984) for 2 h and followed by TGF-β2 treatment for 15 min (**a**) and 48 h (**b**), the protein expressions were examined with Western blot. **c** CCK8 assay of primary pericytes with different treatment. Data are presented as mean ± SD, statistics were performed by one-way ANOVA. $^{**}P < 0.01$, $^{***}P < 0.001$.

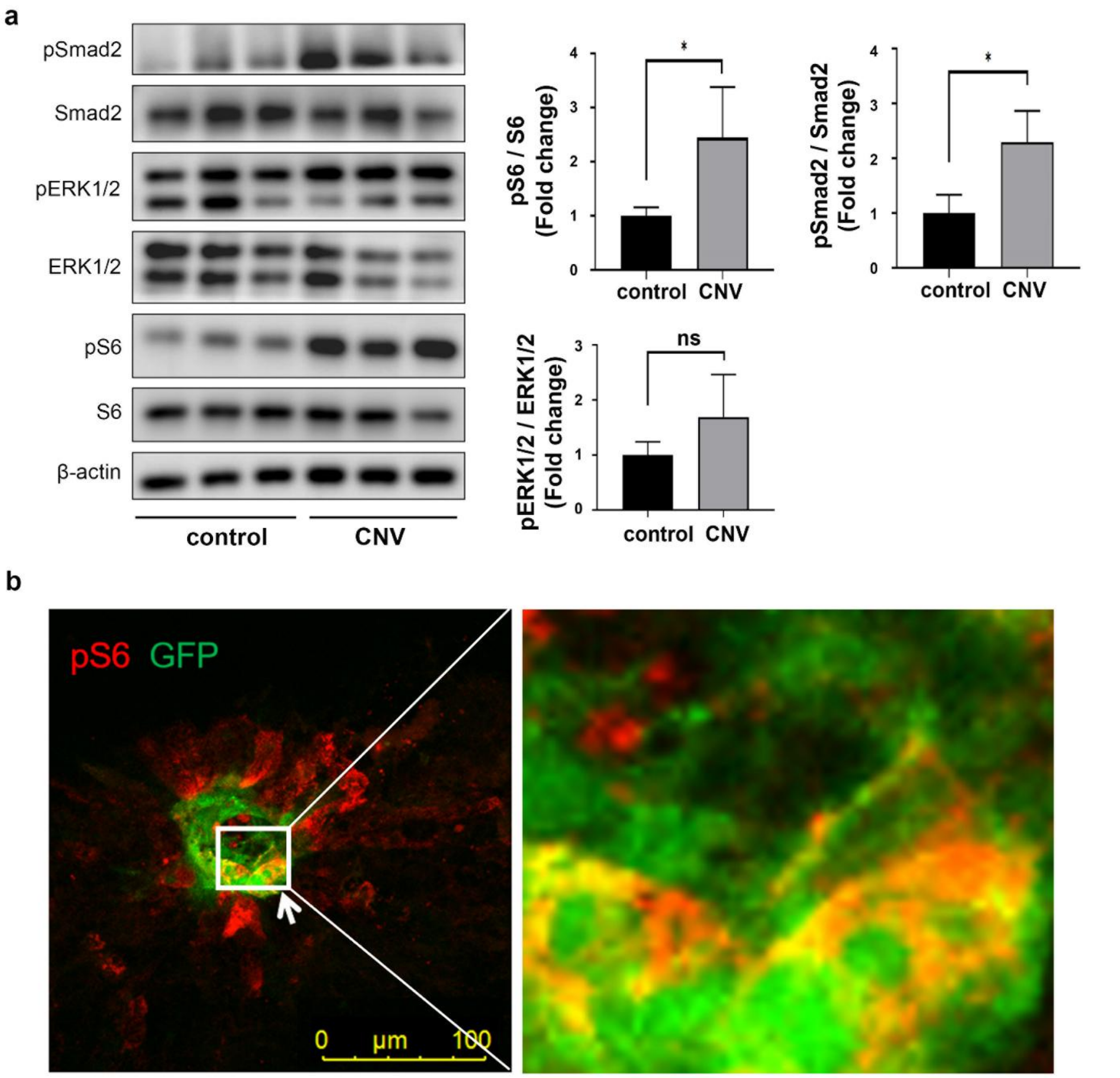

Supplementary Fig.6 mTOR signaling and Smad2 signaling were activated in CNV model. a Western blot of the lysates of the RPE-choroid complex from laser-induced CNV or control mice. **b** Immunofluorescence of pS6 in RPE-choroid flatmount treated with laser injury. Green, GFP; Red, pS6. Scale bar,100 μm. Data are presented as mean ± SD. Statistics were performed by Welch's t test. ns, non-significant difference. *$P < 0.05$

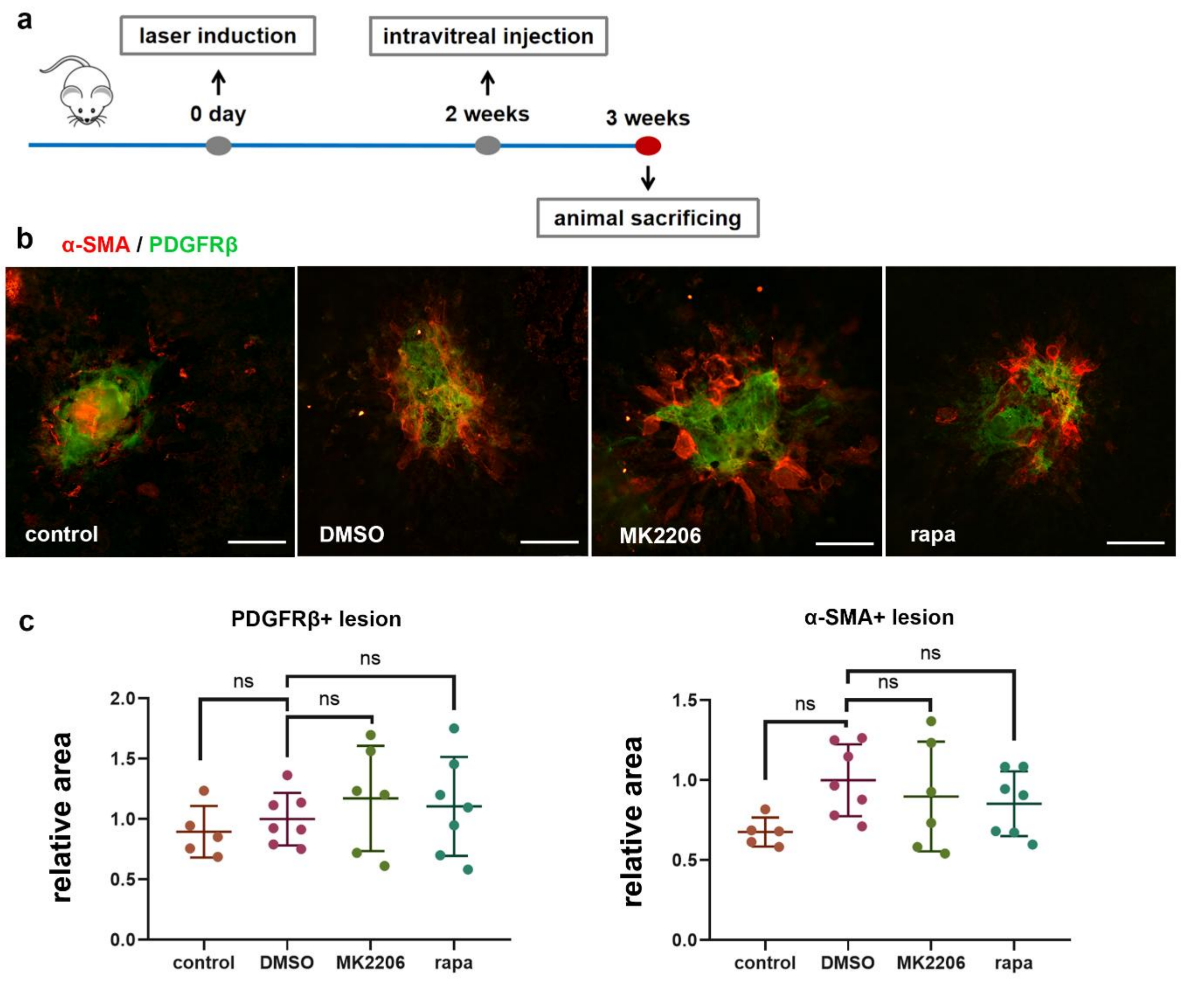

Supplementary Fig.7 Late inhibition of Akt/mTOR had no effect on pericytes proliferation or subretinal fibrosis. a Schematic diagram for laser-induced CNV mouse model establishment and intravitreal injection of the inhibitors. **b** Pericytes infiltration and subretinal fibrosis showed no significant difference among these four groups. MK2206, Akt inhibitor; rapa, mTOR inhibitor; DMSO, control group. Green, PDGFRβ; Red, α-SMA. Scale bar, 100 μm. **c** Quantitative measurements of PDGFRβ-positive areas and α-SMA-positive areas (n = 5-7 per group) in **b**. Data are presented as mean ± SD. Statistics were performed by one-way ANOVA. ns, non-significant difference.
